# Chitosan-based delivery system enhances antimicrobial activity of chlorhexidine

**DOI:** 10.3389/fmicb.2022.1023083

**Published:** 2022-09-29

**Authors:** Lisa Myrseth Hemmingsen, Pimmat Panchai, Kjersti Julin, Purusotam Basnet, Mona Nystad, Mona Johannessen, Nataša Škalko-Basnet

**Affiliations:** ^1^Drug Transport and Delivery Research Group, Department of Pharmacy, University of Tromsø The Arctic University of Norway, Tromsø, Norway; ^2^Research Group for Host-Microbe Interaction, Department of Medical Biology, University of Tromsø The Arctic University of Norway, Tromsø, Norway; ^3^Women’s Health and Perinatology Research Group, Department of Clinical Medicine, University of Tromsø The Arctic University of Norway, Tromsø, Norway; ^4^IVF Clinic, Women’s Clinic, University Hospital of North Norway, Tromsø, Norway

**Keywords:** chitosan, chlorhexidine, lipid-based vesicles, membrane-active antimicrobials, skin wound healing, bioactive polymer, antibacterial activity

## Abstract

Infected chronic skin wounds and other skin infections are increasingly putting pressure on the health care providers and patients. The pressure is especially concerning due to the rise of antimicrobial resistance and biofilm-producing bacteria that further impair treatment success. Therefore, innovative strategies for wound healing and bacterial eradication are urgently needed; utilization of materials with inherent biological properties could offer a potential solution. Chitosan is one of the most frequently used polymers in delivery systems. This bioactive polymer is often regarded as an attractive constituent in delivery systems due to its inherent antimicrobial, anti-inflammatory, anti-oxidative, and wound healing properties. However, lipid-based vesicles and liposomes are generally considered more suitable as delivery systems for skin due to their ability to interact with the skin structure and provide prolonged release, protect the antimicrobial compound, and allow high local concentrations at the infected site. To take advantage of the beneficial attributes of the lipid-based vesicles and chitosan, these components can be combined into chitosan-containing liposomes or chitosomes and chitosan-coated liposomes. These systems have previously been investigated for use in wound therapy; however, their potential in infected wounds is not fully investigated. In this study, we aimed to investigate whether both the chitosan-containing and chitosan-coated liposomes tailored for infected wounds could improve the antimicrobial activity of the membrane-active antimicrobial chlorhexidine, while assuring both the anti-inflammatory activity and cell compatibility. Chlorhexidine was incorporated into three different vesicles, namely plain (chitosan-free), chitosan-containing and chitosan-coated liposomes that were optimized for skin wounds. Their release profile, antimicrobial activities, anti-inflammatory properties, and cell compatibility were assessed *in vitro*. The vesicles comprising chitosan demonstrated slower release rate of chlorhexidine and high cell compatibility. Additionally, the inflammatory responses in murine macrophages treated with these vesicles were reduced by about 60% compared to non-treated cells. Finally, liposomes containing both chitosan and chlorhexidine demonstrated the strongest antibacterial effect against *Staphylococcus aureus*. Both chitosan-containing and chitosan-coated liposomes comprising chlorhexidine could serve as excellent platforms for the delivery of membrane-active antimicrobials to infected wounds as confirmed by improved antimicrobial performance of chlorhexidine.

**GRAPHICAL ABSTRACT:**
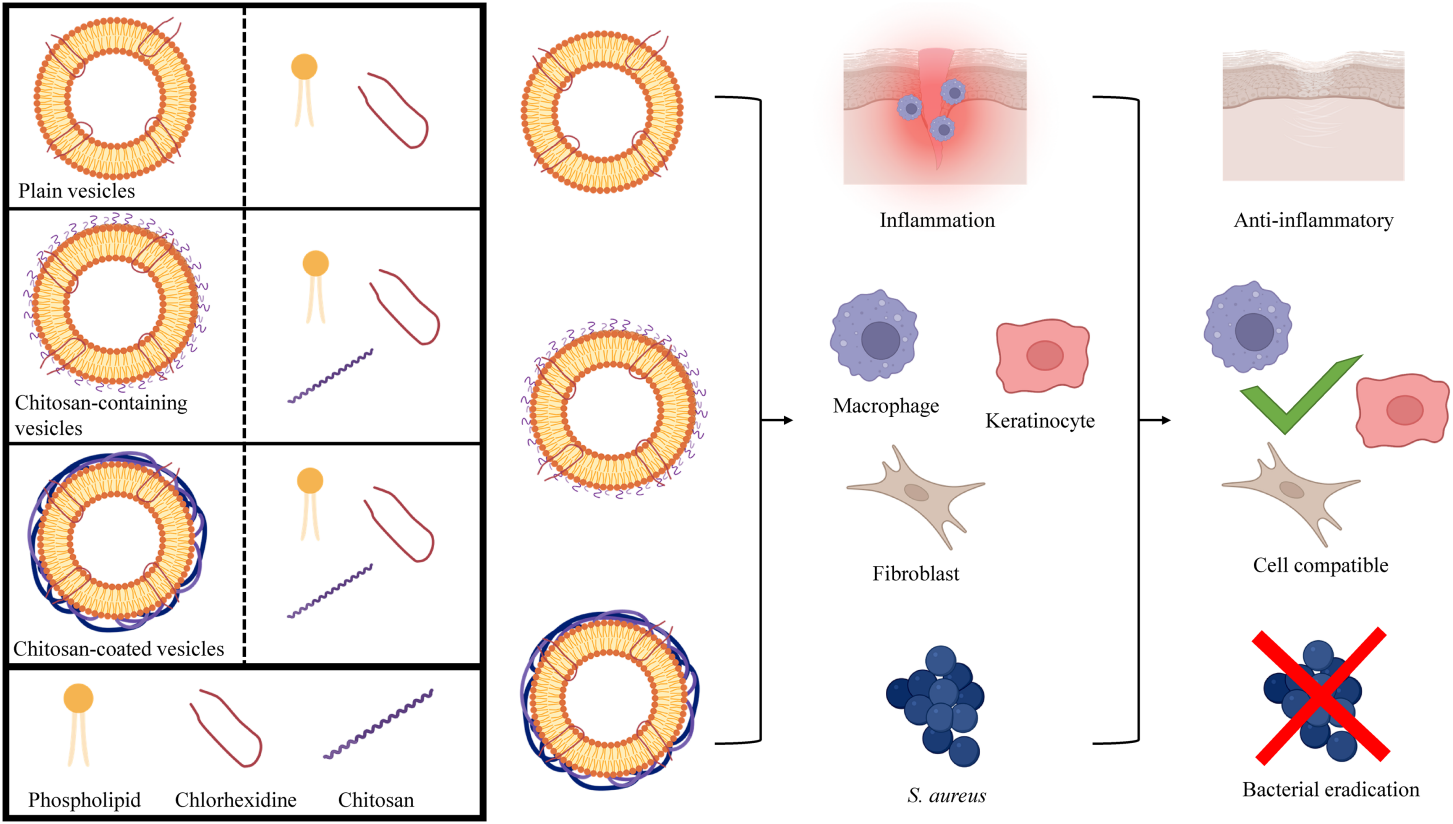

## Introduction

Skin wounds, and particularly chronic wounds, are placing an enormous strain on health care systems worldwide; in 2018 the prevalence of chronic wounds was estimated to be approximately 1–2% in the general population (Kaiser et al., 2021). There is a solid consensus that one of the most important factors permitting wounds to heal properly is the ability to lower the microbial burden and inflammation in the wound bed (Eriksson et al., 2022). However, the rising antimicrobial resistance (AMR) and bacteria’s production of biofilms are making this undertaking more challenging, therefore innovative strategies are urgently needed to mend the situation (Barrigah-Benissan et al., 2022). In this scenario, chitosan could play an important role both because of its inherent biological properties, but also its ability to improve efficacy of antimicrobial compounds (Hemmingsen et al., 2021a). Chitosan is among the most frequently used polymers in pharmaceutical technology and drug delivery systems (Pramanik and Sali, 2021). The interest in chitosan emanates from its many beneficial attributes, such as antimicrobial, anti-inflammatory, anti-oxidative, and hemostatic properties (Iacob et al., 2021). Additionally, this polymer, derived from deacetylated chitin found in crab, shrimp, krill shells, and fungi, is biodegradable and biocompatible with generally low toxicity (Bakshi et al., 2020). Numerous studies have confirmed its potential in skin therapy, especially against skin infections (Hemmingsen et al., 2021c). However, lipid-based systems are more frequently used in skin delivery; liposomes are often considered attractive because of their ability to closely interact with the skin structure (Matei et al., 2021). Additionally, liposomes and lipid-based vesicles provide prolonged release, protect the entrapped antimicrobial, and allow high local drug concentrations at the infected site (Nwabuife et al., 2021). To utilize the advantageous attributes from both lipid-based systems and chitosan, they can be combined, as, e.g., in chitosomes (chitosan-containing liposomes) with chitosan on the surface and in the interior of the liposomes or chitosan-coated liposomes (Sebaaly et al., 2021). These vesicles have been investigated for several applications, however, mainly for mucosal delivery (Sebaaly et al., 2021). Additionally, their role in wound healing has also been investigated (Mengoni et al., 2017; Eid et al., 2022), yet their role in antimicrobial wound therapy is not fully explored. We propose that by tailoring chitosan’s availability on vesicle surface we could improve the antimicrobial potential of chitosan-comprising vesicles for wound therapy.

Taking advantage of the antimicrobial properties and potentially elevate the effect of chitosan, chitosan-containing or chitosan-coated drug delivery systems could be further combined with membrane-active antimicrobials (MAAs); their combination could generate a synergetic antimicrobial effect (Hemmingsen et al., 2021b). Among antiseptics that are often used to treat skin and soft tissue infections, the MAA chlorhexidine (CHX), is one of the most common (Hoang et al., 2021). Its main mechanism of action is proposed to be a destruction of the bacterial membranes; however, precipitation of the cytoplasm has been observed at higher doses (Hubbard et al., 2017). Unfortunately, studies show growing resistance towards CHX which might affect its future effectiveness in the clinics (Fritz et al., 2013; Cieplik et al., 2019; Abdel-Sayed et al., 2020). Here, the drug delivery systems could play a valuable role. Carefully tailored delivery systems could improve the antimicrobial efficacy of antimicrobial compounds by increasing their local concentration and retention time, protect antimicrobial compounds, and improve interaction with bacterial membranes (Osman et al., 2022). Furthermore, in chronic wounds, the additional beneficial biological properties of chitosan could improve wound healing by directly affecting the healing cascade or reducing inflammation and oxidative radicals (Iacob et al., 2021).

In our previous study, we investigated the effect of medium molecular weight (M_w_) chitosan combined with liposomes on inflammatory responses and antimicrobial potential (Hemmingsen et al., 2021b). In the current study, we assessed whether the insertion of chitosan into lipid vesicles, as in chitosan-containing liposomes or chitosomes, or chitosan-coating of pre-made lipid carriers, influenced the CHX release and biological properties of the novel system. Furthermore, we investigated the ability of low M_w_ chitosan to improve the anti-inflammatory and antimicrobial properties of CHX. The antimicrobial activity of chitosan is not fully elucidated, however, the most common explanations for its antimicrobial properties are proposed to be linked to the interaction between positively charged chitosan and the slightly negatively charged bacterial membrane (Khan et al., 2020; Xia et al., 2022). However, chitosan’s biological properties are coupled to its M_w_ and degree of deacetylation. Chitosans of higher M_w_ are proposed to form an envelope around the bacterial membrane, limiting nutrient uptake and growth, while chitosans of lower M_w_ are more prone to penetrate the bacterial membrane and interact with intracellular components (Matica et al., 2019). We aimed to exploit the latter mechanism to improve the antimicrobial potential of CHX.

## Materials and methods

### Materials

Chitopharm™ S-Chitosan with low M_w_ (50–1000 kDa), degree of deacetylation >70% was kindly provided by Chitinor (Tromsø, Norway). Lipoid S100 was kindly provided by Lipoid GmbH (Ludwigshafen, Germany). Methanol ≥99.9%, HiPerSolv CHROMANORM® for LC–MS, phosphate buffered-saline (PBS, pH 7.4) tablets and acetic acid glacial were procured from VWR International (Fontenay-sous-Bois, France). Chlorhexidine ≥99.5%, glycerol solution (86–89%), glycine hydrochloride ≥99% (HPLC), sodium chloride, hydrochloric acid, Cell Counting Kit-8 (CCK-8), and Kollisolv® polyethylene glycol (PEG) E 400 were obtained from Sigma-Aldrich (St. Louis, MO, United States). Cibacron Brilliant Red 3BA was a product from Santa Cruz Biotechnology (Dallas, TX, United States). Ortho-phosphoric acid ≥85% was purchased from Kebo Lab Ab (Oslo, Norway). Penicillin–streptomycin and Roswell Park Memorial Institute (RPMI) medium 1640 were purchased from Sigma-Aldrich (Steinheim, Germany). Lipopolysaccharide (LPS, from *Escherichia coli* 055:B5), sulfanilamide ≥98% and N-(1-Naphthyl)ethylenediamine dihydrochloride ≥98% were obtained from Sigma Life Science Norway AS (Oslo, Norway). Dulbecco’s Modified Eagle Medium high glucose w/ l-glutamine (DMEM-hg) and sodium pyruvate and fetal bovine serum (FBS) were purchased from Biowest (Nuaillé, France). Blood agar plates, saline solution, and Mueller–Hinton broth were supplied by University Hospital of North Norway (Tromsø, Norway). Murine macrophage RAW 264.7 cells were ordered from ATCC (Manassas, VA, United States). Human Dermal Fibroblasts, Neonatal (NHDF-neo) were obtained from Lonza (Basel, Switzerland), and HaCaT cell line (immortalized human keratinocytes) from CLS Cell Lines Service GmbH (Eppelheim, Germany). *Staphylococcus aureus* (ATCC® BAA-1721™) MSSA476 was ordered from LGC standards AB (Borås, Sweden).

### Preparation of vesicles

#### Preparation of plain lipid carriers or chitosan-containing liposomes

Vesicles were produced by the thin film method as described previously (Shukla et al., 2020; Hemmingsen et al., 2021a). In brief, Lipoid S100 (200 mg) and CHX (10 mg) were dissolved in methanol; the solvent was removed by evaporation (Büchi rotavapor R-124 with vacuum controller B-721, Büchi Vac® V-500, Büchi Labortechnik, Flawil, Switzerland) at 60 mBar and 45°C for at least 1 h. The lipid film was dislodged with 10 ml distilled water to create plain (chitosan-free) lipid carriers or 10 ml 0.2% (w/v) chitosan solution in 0.1 M acetic acid, to form chitosan-containing liposomes. Both formulations were shaken to anneal vesicles. Empty lipid carriers were prepared in the same manner without CHX present. The vesicles were stored in the refrigerator (4°C) prior to size reduction.

#### Vesicle coating with chitosan

**Figure 1:**
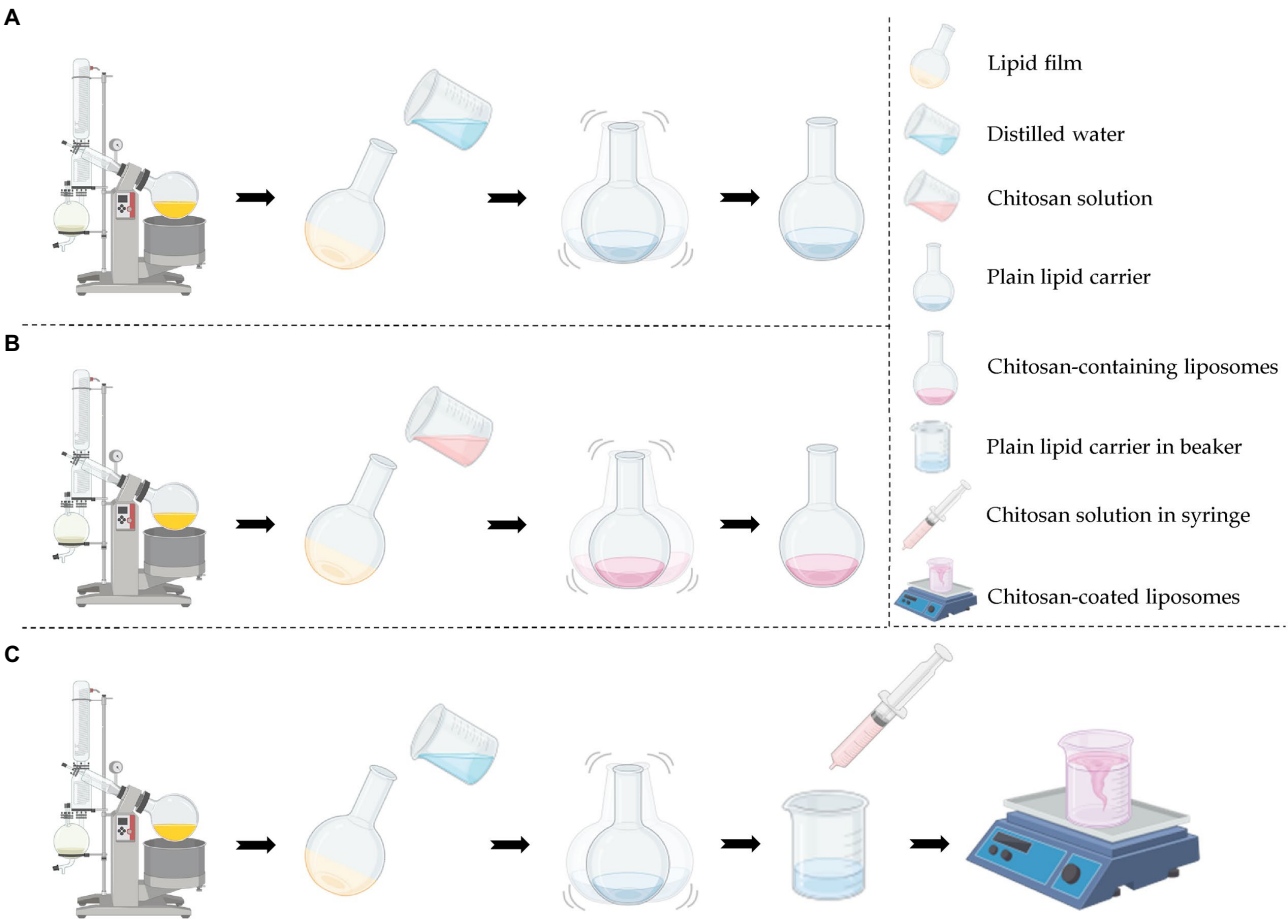
Production procedure of the different vesicles. **(A)** Production of plain lipid carrier with or without chlorhexidine. **(B)** Production of chitosan-containing liposomes with or without chlorhexidine. **(C)** Production of chitosan-coated liposomes with or without chlorhexidine. Created with BioRender.com.

Plain lipid carriers with or without CHX were coated with 0.2% (w/v) chitosan solution in 0.1 M acetic acid (1:1, v/v, Jøraholmen et al., 2014). The chitosan solution was added drop-wise (1.22 min/ml) under continuous stirring (250 rpm). The suspensions were stirred for another hour at 24°C before refrigeration (4°C). The lipid and chitosan concentrations were adjusted to be comparable prior to all further experiments. The production of the different vesicles is depicted in Figure 1.

#### Vesicle size reduction

The size of the vesicles was reduced using probe sonication and manual extrusion. The amplitude of the probe (SONICS high intensity ultrasonic processor, 500-watt model, 13 mm probe diameter, Sonics & Materials Inc., Newtown, CT, United States) was set to 40% and the samples were kept on ice bath to avoid extensive heating. Extrusion was performed with polycarbonate membranes (Nuclepore Track-Etch Membrane, Whatman House, Maidstone, United Kingdom) with average pore size of 0.4 μm (Cauzzo et al., 2020). The size of the different formulations was reduced as described in Table 1 to attain vesicles of similar sizes.

**Table 1:** Vesicle type, designation, and size reduction procedure.

	**Sonication time (s)**	**Sonication intervals**	**Rounds of extrusion**
PL	10	10	3
PL-CHX	5	1	–
CH	10	10	3
CH-CHX	5	2	–
CO	10	18	–
CO-CHX	5	1	–

PL, Plain, empty lipid carrier; PL-CHX, Plain CHX-lipid carrier; CH, Chitosan-containing empty liposomes; CH-CHX, Chitosan-containing CHX-liposomes; CO, Chitosan-coated empty liposomes; CO-CHX, Chitosan-coated CHX-liposomes.

### Vesicle characterization

#### Vesicle size and zeta potential measurements

The size of vesicles was measured with NICOMP Submicron particle sizer (NICOMP Particle Sizing System, Santa Barbara, CA, United States) at an intensity of 250–350 kHz reached by dilution in filtered (0.2 μm) distilled water (Hemmingsen et al., 2021b). The vesicles were measured in three rounds of 15–20 min (to attain stable readings) at 22–24°C, and the weight-intensity distribution was recorded (as cumulative size of 80% of the population).

The zeta potential was measured with Zetasizer Nano Zen 2,600 (Malvern, Worcestershire, United Kingdom). The samples were diluted in filtered (0.2 μm) tap water (assuring counter ions) to an appropriate concentration (according to attenuation) and measured at 25°C in three cycles using a DTS1070 cell (Malvern, Worcestershire, United Kingdom, Jøraholmen et al., 2015).

The pH of vesicle suspensions was measured using sensION+ PH31 pH benchtop meter (Hach, Loveland, CO, United States).

#### Entrapment efficiency

Unentrapped CHX was removed from the vesicle suspension using dialysis tubing with M_w_ cut-off 12–14 kDa (Spectra/Por®4, Spectrum®, VWR International, Fontenay-sous-Bois, France). An aliquot of 1 ml of vesicle suspension was dialyzed against 1 l distilled water under stirring for 4 h at room temperature. The CHX incorporated in the liposomes was quantified using Spark M10 multimode plate reader (Tecan Trading AG, Männedorf, Switzerland) at 261 nm (Hemmingsen et al., 2021a).

### Surface-available chitosan determination

Quantification of surface-available chitosan was performed as previously described (Muzzarelli, 1998; Jøraholmen et al., 2015). In short, glycine buffer (250 ml, pH 3.2) was prepared in distilled water using 1.87 g glycine and 1.46 g NaCl. This buffer (81 ml) was further diluted to a total volume of 100 ml in 0.1 M HCl. To quantify chitosan, a dye solution was prepared. An aliquot of 150 mg Cibacron Brilliant Red 3B-A was dissolved in distilled water (100 ml). The glycine buffer was used to dilute 5 ml of the dye solution to a total volume of 100 ml. An aliquot of 300 μl of diluted vesicle suspensions (distilled water, 1:1, v/v) were mixed with 3 ml of the diluted Cibacron dye and surface-available chitosan was quantified using Spark M10 multimode plate reader (Tecan Trading AG, Männedorf, Switzerland) at 575 nm (Jøraholmen et al., 2015).

### Vesicle stability

The stability of the vesicles was evaluated after 2- and 4-week storage at 4°C. The parameters evaluated were the vesicle size, zeta potential, and pH as described in the section Vesicle size and zeta potential measurements.

### *In vitro* chlorhexidine release

*In vitro* CHX release studies were performed using a Franz cell diffusion system (PermeGear, Hellertown, PA, United States). Pre-soaked cellophane membranes (Max Bringmann KG, Wendelstein, Germany) were used as diffusion barriers with area of 1.77 cm^2^ (Jøraholmen et al., 2014). Due to the low water solubility of CHX base (Farkas et al., 2001), the acceptor chamber was filled with PEG E 400 (10%, v/v) in distilled water (12 ml acceptor volume, Hemmingsen et al., 2021b). The temperature was maintained at 32°C with heated circulating water. Vesicle suspensions (600 μl) were added to the donor chamber. Samples were withdrawn after 1, 2, 3, 4, 5, 8, and 24 h, and the sample volume was replaced with fresh medium to maintain sink conditions. The release from vesicles was compared to non-formulated CHX (dissolved in release media). Quantitative analysis was carried out using Spark M10 multimode plate reader (Tecan Trading AG, Männedorf, Switzerland) at 261 nm (Hemmingsen et al., 2021b).

### Evaluation of cell viability and anti-inflammatory responses

#### Assessment of cell viability

Assessment of cell viability was accomplished using the CCK-8 kit according to methods previously described (Hemmingsen et al., 2021b). The cells (HaCaT; Cauzzo et al., 2020, NHDF-neo; Domiński et al., 2022, and murine macrophages RAW 264.7; Basnet et al., 2012; Cauzzo et al., 2020) in complete RPMI medium [containing 10% (v/v) FBS and penicillin–streptomycin; RAW 264.7] or complete DMEM-hg (HaCaT and NHDF-neo) were plated on 96-well plates (90 μl, 1 × 10^5^ cells/ml) and incubated (37°C, 5% CO_2_) for 24 h. Diluted vesicle suspensions (10 μl) were added to the wells (final lipid concentration of 1, 10, and 50 μg/ml) and the plates incubated for another 24 h (37°C, 5% CO_2_). Next, an aliquot of 10 μl CCK-8 reagent was added to each well and the plates were incubated for 4 h. The cell viability was measured using Spark M10 multimode plate reader (Tecan Trading AG, Männedorf, Switzerland) at 450 nm with the reference set to 650 nm. Treated cells were compared to non-treated cells (only complete RPMI or DMEM-hg).

#### Anti-inflammatory activity

The anti-inflammatory activity of the vesicles was assessed by inducing nitric oxide (NO) production in murine macrophages using LPS as previously described (Schulte-Werning et al., 2021). RAW 264.7 cells (Basnet et al., 2012) in complete RPMI medium [containing 10% (v/v) FBS and penicillin–streptomycin] were plated on 24-well plate (1,000 μl, 5 × 10^5^ cells/ml) and incubated (37°C, 5% CO_2_) for 24 h. The complete medium was aspirated and LPS (1 μg/ml, 990 μl) in complete RPMI added to each well. Next, diluted vesicle suspensions (10 μl) were added to the wells at final lipid concentration of 1, 10, and 50 μg/ml, and the plates incubated for another 24 h (37°C, 5% CO_2_). The NO production was assessed by mixing the cell medium and Griess reagent [1:1, v/v; 2.5% phosphoric acid with 1% sulphanilamide and 0.1% N-(−1-naphthyl)ethylenediamine] and analyzing the mixture with Spark M10 multimode plate reader (Tecan Trading AG, Männedorf, Switzerland) at 560 nm. Only complete medium or LPS (1 μg/ml) in complete RPMI served as controls. The LPS-induced cells treated with vesicles were compared to non-treated LPS-induced cells (100%).

### Antimicrobial evaluation

The broth microdilution method was utilized to evaluate the antibacterial properties of the vesicles with or without CHX (Balouiri et al., 2016). Overnight cultures of *S. aureus* MSSA476 were diluted in saline solutions (0.85%, w/w) to a turbidity of 0.5 McFarland; these bacterial suspensions were further diluted (1:150, v/v) in Mueller-Hinton broth. Vesicle suspensions were 2-fold diluted with Mueller-Hinton broth in 96-well plates and the diluted bacterial suspensions added (1:1, v/v). The plates were incubated at 37°C with shaking (100 rpm) for 24 h. Non-treated or treated (with different vesicles) bacteria in suspensions were serially diluted (10-fold) in PBS, plated on blood agar plates and incubated at 37°C overnight. The colony-forming units (CFUs) were counted to evaluate the activity of the tested formulations as compared to non-treated bacteria. Lipid concentrations of 0.3125 mg/ml were used to compare the different vesicle formulations (Ternullo et al., 2019).

### Statistical analyses

The results are generally expressed as means ± SD. Statistical significance was evaluated by student’s *t*-test or one-way ANOVA followed by Turkey’s correction (*p* at least 0.05). All statistical analyses were performed in GraphPad Prism version 9.3.1 for Windows (GraphPad Software LLC, San Diego, CA, United States).

## Results and discussion

### Vesicle characteristics

Size is an important parameter in the development of drug delivery systems; considering the dermal administration route it has been proposed that size around 300 nm might be beneficial assuring that the vesicles are able to reach the deeper layers of the skin without advancing too deep (du Plessis et al., 1994). In our previous studies, we have shown that the vesicles in a size range between 250 and 350 nm provide good eradication of common skin pathogens (Hemmingsen et al., 2021a,b). Furthermore, reports indicate that nanoparticles smaller than 350 nm can diffuse through biofilm pores (Makabenta et al., 2021). Therefore, we aimed for the size range of 250–350 nm for our plain and chitosan-comprising formulations (Table 2). We assessed the vesicle size as cumulative size of 80% of the vesicle populations since some of the vesicles exhibited the bi- or multi-modal distributions that were difficult to directly compare. Most vesicles were slightly over 300 nm in diameter; however, the chitosan-coated liposomes displayed a larger size likely due to the coating procedure. Even though the optimal polydispersity index (PI) is suggested to be about 0.3 for lipid-based vesicles destined for skin delivery (Danaei et al., 2018), our vesicles had a PI below 0.4 and that was deemed acceptable. The vesicle size of chitosan-coated liposomes is often larger and harder to control as compared to non-coated liposomes (Jøraholmen et al., 2015).

**Table 2:** Chitosan-containing liposomes and chitosan-coated liposomes characteristics: mean diameter (≤80%, nm), polydispersity index (PI), zeta potential, entrapment efficacy (EE%), and pH in aqueous medium.

	**Size (**≤**80%, nm)**	**PI**	**Zeta potential (mV)**	**EE%**	**pH**
PL	308 ± 22	0.37 ± 0.04	−1.6 ± 1.4	–	5.8 ± 0.5
PL-CHX	305 ± 14	0.38 ± 0.03	42.9 ± 5.9	63.2 ± 4.8	8.5 ± 0.1
CH	303 ± 18	0.32 ± 0.01	12.4 ± 0.4	–	3.6 ± 0.0
CH-CHX	300 ± 24	0.34 ± 0.07	94.9 ± 2.2	65.7 ± 4.8	3.7 ± 0.0
CO	325 ± 23	0.35 ± 0.01	13.0 ± 0.4	–	3.7 ± 0.0
CO-CHX	393 ± 23	0.39 ± 0.02	83.3 ± 3.1	70.4 ± 3.9	3.8 ± 0.0

Results of size measurements are expressed as means of cumulative size ≤ 80% of vesicle populations (weight-intensity distribution) with their respective SD, while the rest of the results are expressed as means with their respective SD (*n* = 3). PL, Plain, empty lipid carrier; PL-CHX, Plain CHX-lipid carrier; CH, Chitosan-containing empty liposomes; CH-CHX, Chitosan-containing CHX-liposomes; CO, Chitosan-coated empty liposomes; and CO-CHX, Chitosan-coated CHX-liposomes.

Tailoring vesicles with chitosan have previously shown to increase the zeta potential of the delivery system (Mady et al., 2009; Park et al., 2014). The increase in surface charge is an indication of successful addition (coating or insertion) of chitosan indicating that chitosan is available on the surface of the vesicles (Jøraholmen et al., 2014). Additionally, nanoparticles with a cationic character are able to distribute within the biofilm after penetration into the matrix (Makabenta et al., 2021). The surface charge increased even more upon incorporation of CHX in the formulations; the fact that both chitosan and CHX are available on the vesicle surface and able to interact with the bacteria is highly encouraging considering antimicrobial potential of novel system. The entrapment of CHX was relatively high; however, lower than the entrapment achieved when utilizing the one-pot method which provided a CHX entrapment efficiency of 74% in chitosomes (Hemmingsen et al., 2021b). Nonetheless, a high entrapment and surface-available chitosan and CHX are assuring features for successful antimicrobial therapy. Both compounds are available to interact with the bacteria; moreover, the cationic nature of delivery system will improve the interaction between the vesicles and bacteria since bacterial membranes are slightly negatively changed (Epand and Epand, 2009).

### Surface-available chitosan

To confirm that chitosan was available on the vesicle surface and determine to which extent it was available, we quantified the amount of surface-available chitosan on the vesicles using a colorimetric protocol first described by Muzzarelli (Muzzarelli, 1998). The quantity of surface-available chitosan on the vesicles was found to be rather high for all formulations (Table 3). For the empty, chitosan-coated liposomes the amount was comparable to the study of Jøraholmen et al. (2015). However, for the chitosan-containing liposomes, the amount of chitosan that was available on the surface was greater than the amount achieved for the chitosomes prepared with the one-pot method by Andersen et al. (2015). Additionally, contrary to our previous finding (Hemmingsen et al., 2021b), the addition of CHX seemed to increase the amount of surface-available chitosan on the vesicles; however, it was significant only for the chitosan-coated liposomes. Again, it is important to consider the chitosan origin and its M_w_ when comparing the results. Using a different method, Li et al. reported surface-available chitosan in quantities of up to 89.5% (Li et al., 2009). The surface availability is important not only for potential antimicrobial effects but also considering chitosan’s bioadhesive properties that can be beneficial in wound treatment (Hamedi et al., 2022).

**Table 3:** Surface-available chitosan on chitosan-containing liposomes and chitosan-coated liposomes.

	**Surface-available chitosan (%)** ^**1**^
CH	86.2 ± 16.0
CH-CHX	92.2 ± 3.2
CO	55.1 ± 7.4
CO-CHX	84.4 ± 4.2

Results are expressed as means with their respective SD (*n* = 3). CH, Chitosan-containing empty liposomes; CH-CHX, Chitosan-containing CHX-liposomes; CO, Chitosan-coated empty liposomes; and CO-CHX, Chitosan-coated CHX-liposomes.

1 Percentage of initial chitosan concentration.

### Vesicle stability

To assess the vesicle stability the size, PI, zeta potential, and pH of each formulation were evaluated after 2 and 4 weeks of storage at 4°C (Table 4). The size of all vesicles was relatively stable over the 4-week period; however, plain CHX-lipid carriers exhibited a small decrease in size between production and week 2 (*p* = 0.0109) that was not considered as an issue. Furthermore, the size did not change significantly between week 2 and 4; probably due to the surface charge (above 40 mV) and its stabilizing effect. All other parameters remained stable for the entire period. The addition of chitosan to liposomal formulations is often considered to improve the stability of the suspensions, both as a physical measure to maintain the integrity of the bilayers and due to electrostatic effects; however, this seems to be affected by the chitosan concentration (Sebaaly et al., 2021).

**Table 4:** Chitosan-containing liposomes and chitosan-coated liposomes stability after 2 and 4 weeks of storage: mean diameter (≤80%, nm), polydispersity index (PI), zeta potential, and pH in aqueous medium.

	**Week**	**Size (80%, nm)**	**PI**	**Zeta potential (mV)**	**pH**
PL	2	326 ± 54	0.44 ± 0.05	−2.6 ± 0.6	6.0 ± 0.2
	4	298 ± 25	0.39 ± 0.05	−3.9 ± 0.1	5.7 ± 0.3
PL-CHX	2	272 ± 11	0.41 ± 0.02	40.2 ± 7.6	7.7 ± 0.2
	4	259 ± 8	0.40 ± 0.01	42.2 ± 10.5	7.8 ± 0.4
CH	2	307 ± 19	0.32 ± 0.00	11.1 ± 0.9	3.6 ± 0.0
	4	307 ± 24	0.31 ± 0.01	11.1 ± 0.9	3.7 ± 0.0
CH-CHX	2	285 ± 5	0.28 ± 0.01	92.1 ± 7.8	3.8 ± 0.0
	4	280 ± 6	0.29 ± 0.02	91.9 ± 3.3	3.8 ± 0.0
CO	2	316 ± 17	0.36 ± 0.01	12.5 ± 1.0	3.7 ± 0.0
	4	328 ± 25	0.37 ± 0.01	11.8 ± 0.6	3.7 ± 0.0
CO-CHX	2	375 ± 44	0.39 ± 0.02	83.3 ± 3.2	3.8 ± 0.0
	4	371 ± 40	0.42 ± 0.03	78.6 ± 0.9	3.8 ± 0.0

Vesicle stability after 2 and 4 weeks of storage at 4°C. Results of size measurements are expressed as means of cumulative size ≤80% of vesicle populations (weight-intensity distribution) with their respective SD, while the rest of the results are expressed as means with their respective SD (*n* = 3), while the rest of the results are expressed as means with their respective SD (*n* = 3). PL, Plain, empty lipid carrier; PL-CHX, Plain CHX-lipid carrier; CH, Chitosan-containing empty liposomes; CH-CHX, Chitosan-containing CHX-liposomes; CO, Chitosan-coated empty liposomes; and CO-CHX, Chitosan-coated CHX-liposomes.

### *In vitro* chlorhexidine release

**Figure 2:**
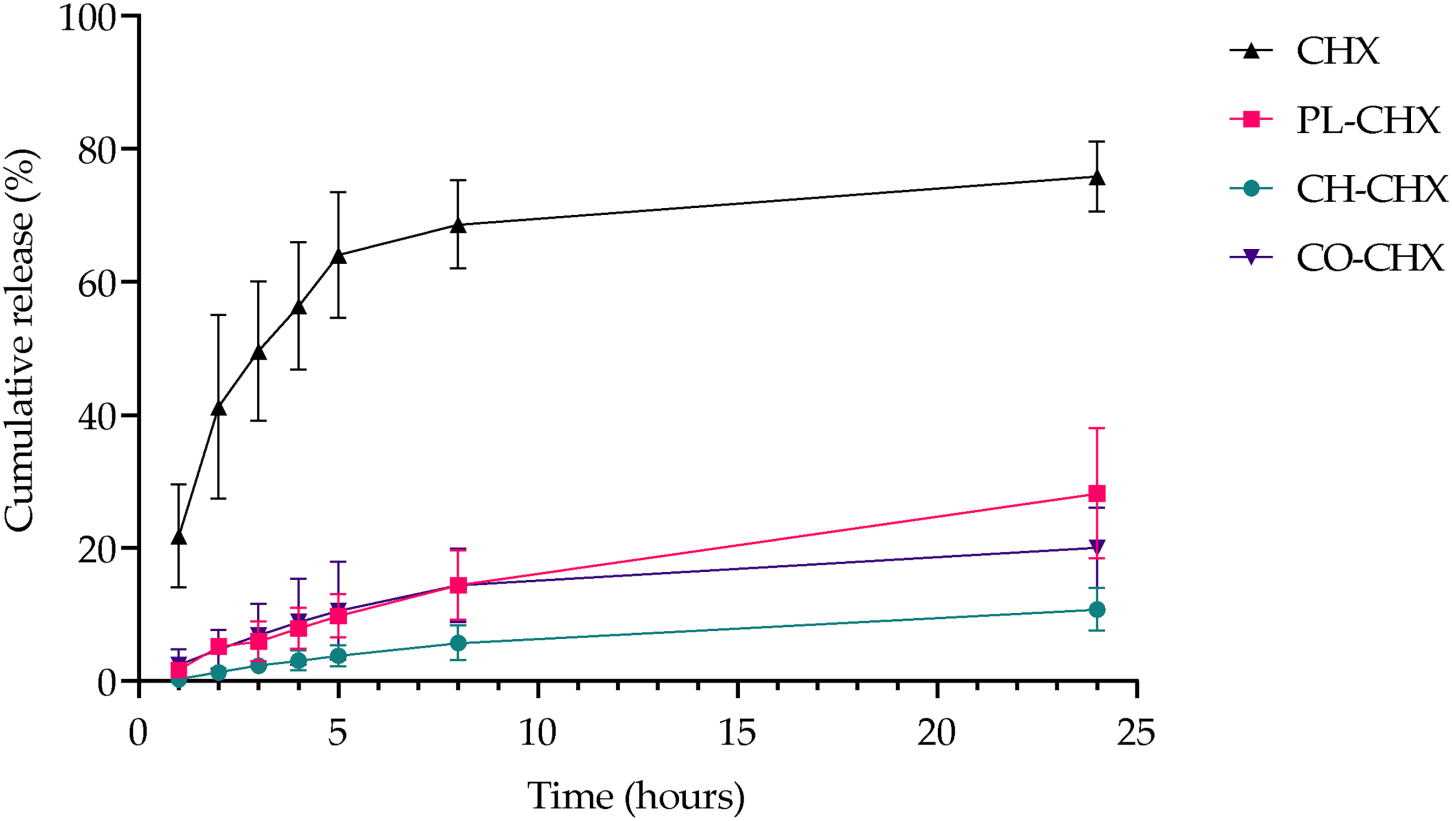
Cumulative *in vitro* release of CHX from non-formulated and formulated CHX over 24 h at 32°C. Results are expressed as release percentage compared to entrapped amount of CHX and means with their respective SD (*n* = 3). Chlorhexidine (CHX), non-formulated CHX in media; PL-CHX, Plain CHX-lipid carrier; CH-CHX, Chitosan-containing CHX-liposomes; and CO-CHX, Chitosan-coated CHX-liposomes.

As a result of the physical presence of chitosan and its physiochemical properties, chitosan could affect the release rate of active compounds from the vesicles (Gibis et al., 2016). Therefore, we investigated the CHX release from the plain lipid carriers, chitosan-containing, and chitosan-coated liposomes (Figure 2). Non-formulated CHX, dissolved in the release medium, was used as a control. After 24 h, the plain CHX-lipid carrier had released significantly more CHX than the chitosan-containing liposomes (*p* = 0.0371). However, no difference in the release was observed between the chitosan-containing and chitosan-coated liposomes. All vesicles significantly decreased the rate of release compared with non-formulated CHX at all time points. This prolonged release profile with gradual, long-lasting release of the compounds is highly beneficial for a drug delivery system intended for topical, antimicrobial therapy. First, these delivery systems could provide a high local concentration important for the therapeutic outcome; however, this is depending on whether the concentration reaches an effective concentration limit (Allen and Cullis, 2013). To prove the effect, biological assays are required. Second, drug delivery systems with prolonged release of the antimicrobial compound could help prevent regrowth of bacteria as well as ensure long-lasting antimicrobial effects (Piras et al., 2015). Third, as the compounds are retained onto/in the skin assuring local depot, the potential for reaching the systemic circulation is limited (Cui et al., 2021). The latter is highly relevant when limiting AMR.

Polymyxin B, another MAA, has previously displayed slower release rate from chitosan-modified liposomes. The vesicles released polymyxin B over a period of 24 h, while the non-formulated polymyxin B was completely released already after 12 h (Fu et al., 2019). On the other hand, Park et al. reported faster permeation rate of the MAA nisin from coated liposomes than uncoated liposomes; however, this study was conducted with mouse skin (Park et al., 2014). It is rather challenging to compare the release data from different studies due to the differences in physicochemical properties of active compounds and experimental settings.

### Cell viability and anti-inflammatory responses

Liposomes and many other lipid-based delivery systems are generally regarded to be highly biocompatible, biodegradable, and safe. Furthermore, the toxicity of certain pharmaceutical compounds is often reduced when they are entrapped in these drug delivery systems (Liu et al., 2022). Nevertheless, the systems’ effect on relevant cells is a critical parameter to be assessed in the development of new carriers or upon entrapment of new pharmaceutical compounds. The safety of these carriers is highly influenced by different features of the systems, such as the composition, size, size distribution, and surface properties (Liu et al., 2022). Consequently, we investigated cell compatibility in relevant cells, namely keratinocytes, fibroblasts, and macrophages, as well as the system’s influence on inflammatory responses in macrophages.

#### Cell viability

**Figure 3:**
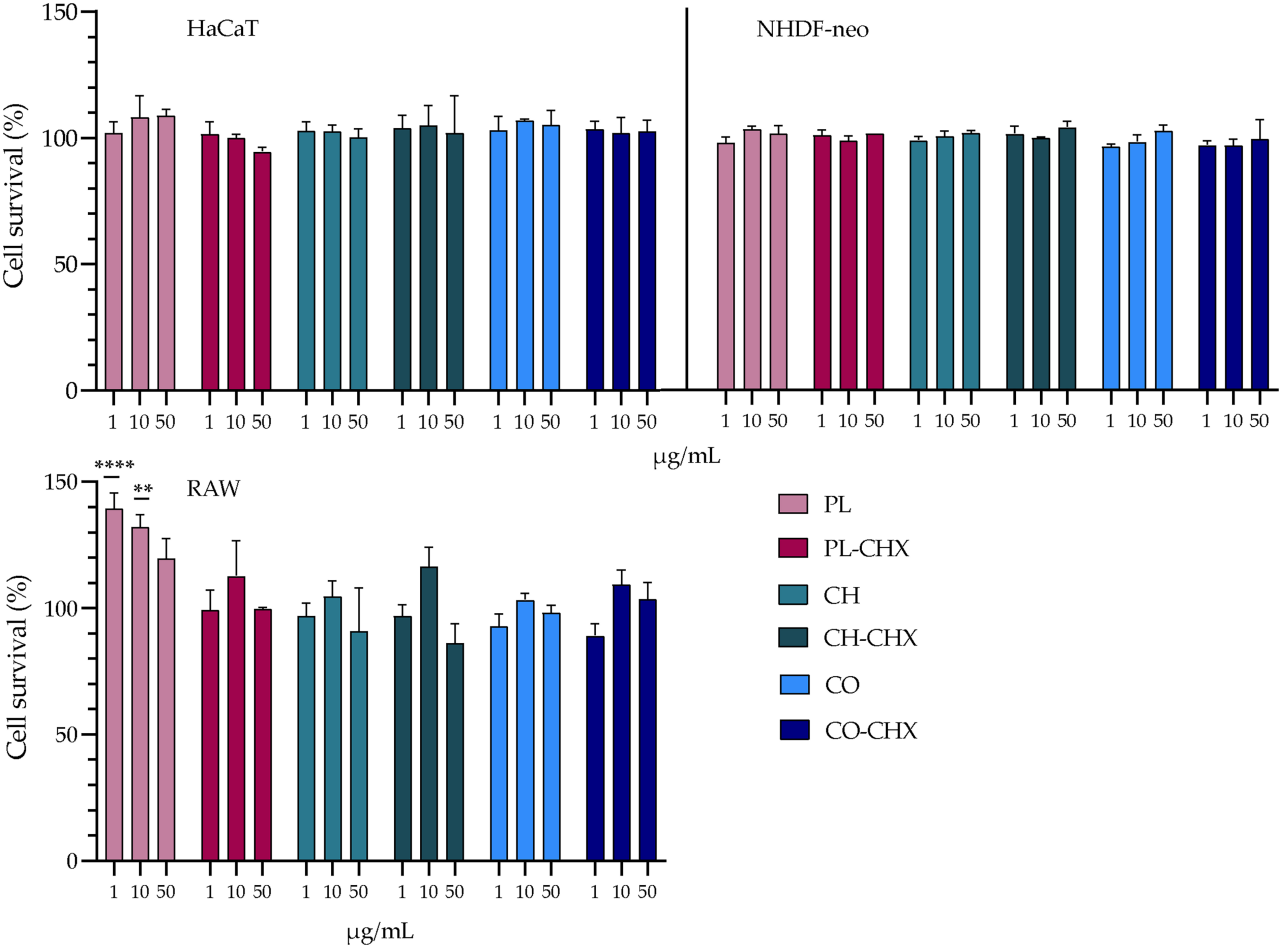
Evaluation of cell toxicity of chitosan-containing liposomes and chitosan-coated liposomes in HaCaT, NHDF-neo cells, and RAW 264.7. Three different concentrations were tested, namely 1, 10, and 50 μg/ml lipid, and the results are presented as cell viability of treated cells compared to control (100%). Control cells were only supplemented with complete medium; the cell viability is thereof considered as 100%. The results are expressed as means with their respective SD (*n* = 3). PL, plain, empty lipid carrier; PL-CHX, plain CHX-lipid carrier; CH, chitosan-containing empty liposomes; CH-CHX, chitosan-containing CHX-liposomes; CO, chitosan-coated empty liposomes; and CO-CHX, chitosan-coated CHX-liposomes. **p ≤ 0.01, ****p ≤ 0.0001.

As previously mentioned, liposomes can reduce the toxicity of pharmaceutical compounds (Nwabuife et al., 2021). Similarly, chitosan is also regarded biocompatible and biodegradable (Rashki et al., 2021). However, it is known that several alternations could change the properties of the materials, especially in the nano-range. For instance, the safety of chitosan is often considered to be linked to its degree of deacetylation and M_w_ (Rashki et al., 2021). The MAA, CHX, has in previous studies displayed toxicity in different cells, e.g., macrophages, keratinocytes, and fibroblasts (Li et al., 2014; Borges et al., 2017). Therefore, the potential toxicity of empty and CHX-loaded vesicles was assessed in these cells (Figure 3). In HaCaT and NHDF-neo cells, the viability of cells was unaffected by the treatment with both empty and CHX-loaded vesicles with or without chitosan. This is highly beneficial, as the viability of these cells is crucial for the successful therapy by therapeutics intended for wounds. Both keratinocytes and fibroblasts play active roles in the inflammatory phase in wounds; their release of cytokines and growth factors maintains hemostasis and influences other cells to participate in the process of wound closure (Wojtowicz et al., 2014). Moreover, keratinocytes are especially important in our defense against bacterial invasion due to their ability to release the antimicrobial peptides with antibacterial, antifungal, and antiviral activities (Chessa et al., 2020).

In the murine macrophages, no negative effects were observed in the treated cells; however, the empty, plain lipid carriers seemed to improve the viability of the cells, suggesting a proliferative effect. At lipid concentrations of 1 and 10 μg/ml, the viability or cell proliferation was significantly improved compared to control (*p* < 0.0001 and 0.0013, respectively). The proliferative effects of liposomes have previously been demonstrated by Ye et al. (2019); however, in significantly higher concentrations than in the current study. Macrophages play several pivotal roles in the wound healing cascade, for instance, cleaning of pathogens and debris from the wound, activation of immune cells, promotion of migration of other cells, such as keratinocytes and fibroblasts, and breaking down the temporary extracellular matrix (Krzyszczyk et al., 2018). Therefore, their presence and retained viability are of high importance. Furthermore, Hilițanu et al. confirmed the biocompatibility of chitosan-coated liposomes containing erythromycin after oral administration in mice. The authors investigated erythrocyte counts, liver enzyme activity, serum urea plasma levels, immunological biomarkers, and histopathological examinations of liver or kidney, and found no significant changes in the mice (Hilițanu et al., 2021).

#### Anti-inflammatory activity

**Figure 4:**
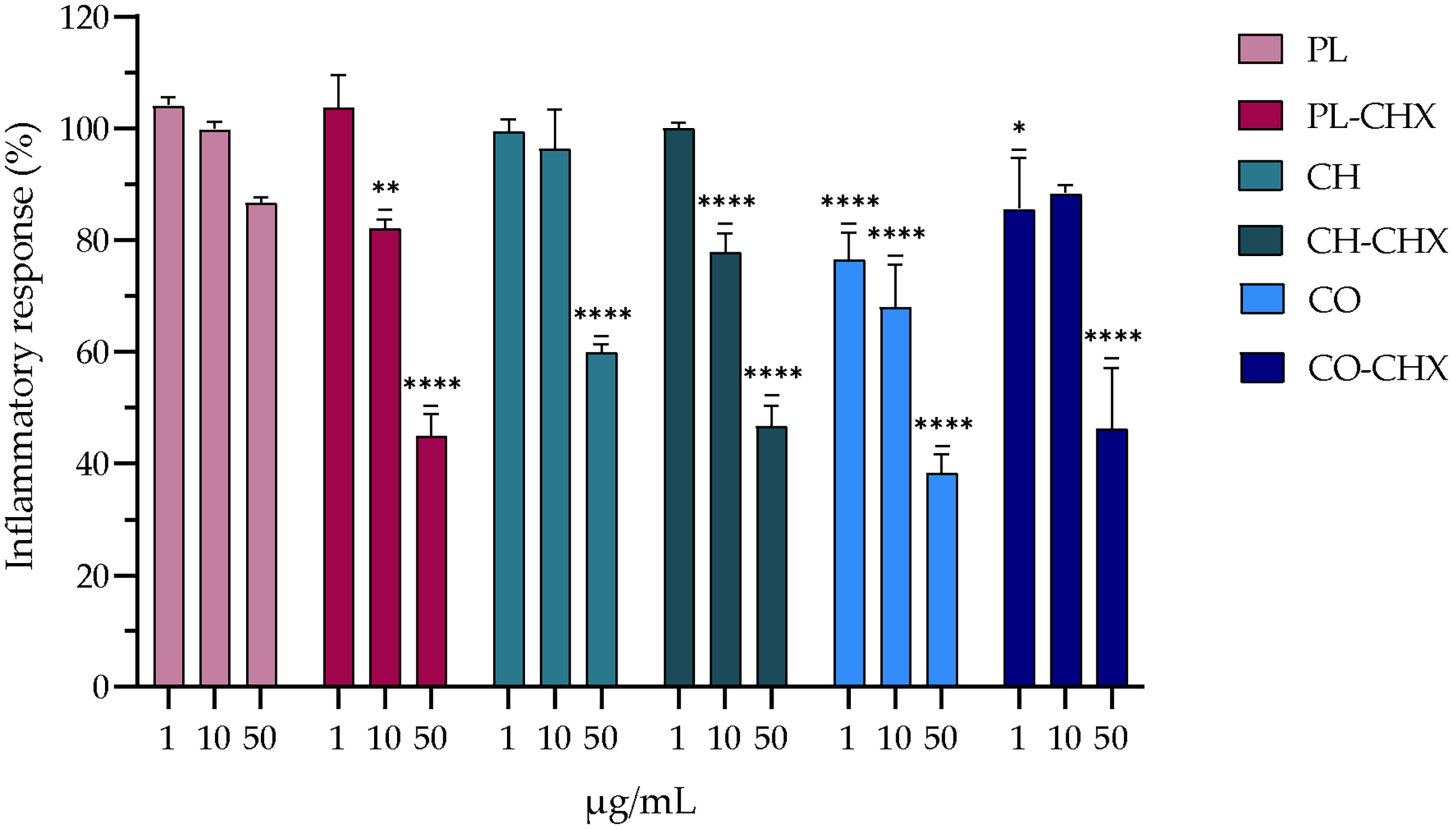
Evaluation of anti-inflammatory activity of chitosan-containing and chitosan-coated liposomes expressed as reduction of nitric oxide (NO) production in RAW 264.7 cells. Three different concentrations were tested, namely 1, 10, and 50 μg/ml lipid, and the results are presented as NO production of treated cells compared to control (100%). Control cells were non-treated lipopolysaccharide (LPS)-induced cells; their production is thereof considered as 100%. The results are expressed as means with their respective SD (*n* = 3). PL, Plain, empty lipid carrier; PL-CHX, Plain CHX-lipid carrier; CH, Chitosan-containing empty liposomes; CH-CHX, Chitosan-containing CHX-liposomes; CO, Chitosan-coated empty liposomes; CO-CHX, Chitosan-coated CHX-liposomes. *p ≤ 0.05, **p ≤ 0.01, ****p ≤ 0.0001, compared to control.

Macrophages bear crucial attributes in wound healing; however, in chronic wounds these cells might also be a part of the problem. Chronic wounds are arrested in a state of inflammation and unable to progress in the healing cascade. This is linked to the presence of pro-inflammatory macrophages (M1-type macrophages) at the site of injury that leads to elevated levels of cytokines and reactive oxygen species, and apoptosis of keratinocytes and fibroblasts (Shamiya et al., 2022). This prolonged state of inflammation is undesirable in wounds as it hinders healing. To evaluate whether novel formulations can act on inflammatory response, we assessed the anti-inflammatory effects in murine macrophages. In macrophages, LPS is recognized by toll-like receptor 4, its binding leading to expression of pro-inflammatory genes. In mice, this leads to overexpression of inducible nitric oxide synthase and subsequent high levels of NO that could serve as an indicator of anti-inflammatory responses. This effect is far greater in mice than in humans, and therefore murine macrophages were utilized to assess the potential anti-inflammatory activity (Krzyszczyk et al., 2018). The results of the inflammatory assessments are presented in Figure 4 and demonstrate a clear dose-dependent anti-inflammatory effect the formulations had on treated cells. The anti-inflammatory activities of the vesicles with chitosan and/or CHX were significantly higher as compared to non-treated LPS-induced macrophages. However, the effects did not seem to be synergetic, namely the presence of both chitosan and CHX did not enhance the effects in synergy. The determined threshold was at about 55–65% reduction. Interestingly, the empty, plain lipid carriers also induced a dose-dependent reduction in inflammatory response; however, this effect was not significant.

### Antimicrobial evaluation

**Figure 5:**
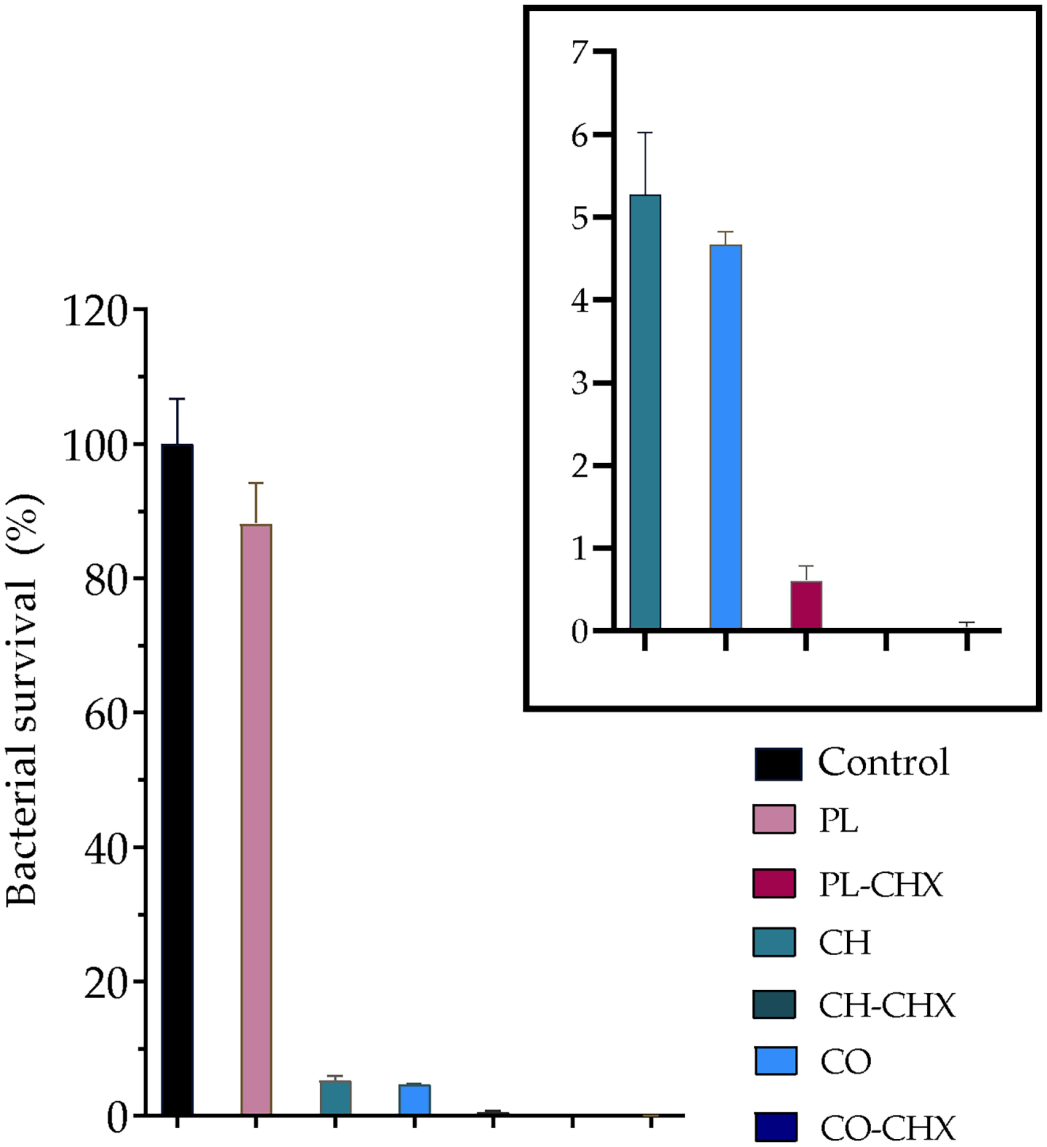
Bacterial survival (%) of *Staphylococcus aureus* MSSA476 (ATCC® BAA-1721™) in the presence of non-formulated and formulated chlorhexidine (CHX) at a lipid concentration of 0.3125 mg/ml. The boxed area is enlarged to depict the chitosan- and/or CHX-containing and/or coated vesicles. The results are expressed as means with their respective SD (*n* = 3). PL, Plain, empty lipid carrier; PL-CHX, Plain CHX-lipid carrier; CH, Chitosan-containing empty liposomes; CH-CHX, Chitosan-containing CHX-liposomes; CO, Chitosan-coated empty liposomes; and CO-CHX, Chitosan-coated CHX-liposomes. ****p ≤ 0.0001, compared to control.

In recent years, more focus has been placed on drug delivery systems and nanostructured materials in the development of new therapeutic options for microbial eradication and prevention. These systems and materials, both organic and inorganic, have demonstrated superior antimicrobial activities against a wide variety of microbial strains (Baranwal et al., 2018). Liposomes are among the most frequently used systems while chitosan has generated interest due to its inherent antimicrobial properties (Baranwal et al., 2018). Considering liposomes, their structure and composition is similar to the bacterial membrane; this could lead to a fusion between liposomes and bacteria resulting in delivery of higher antimicrobial payloads. Additionally, liposomes that possess a positively charged surface could interact with bacteria and further improve the antimicrobial effects (Wang et al., 2020). All of this is expected to improve the therapeutic index and make bacteria more susceptible to antimicrobials associated with delivery system as compared to non-formulated antimicrobials (Wang et al., 2020). To further improve the antimicrobial properties of drug delivery systems, chitosan is often utilized together with other delivery systems. In the current study, we were using an MAA, postulating that chitosan and MAA could act in synergy and enhance the effect on the bacteria. We assessed the antimicrobial activity of our vesicles against *S. aureus*, one of the most common pathogens found in chronic wounds (Alves et al., 2021). As seen in Figure 5, the empty, plain lipid carriers did not display any antimicrobial activity, as expected; however, upon inclusion of chitosan in vesicles, the bacterial survival was dramatically reduced to 5.3 and 4.7% for the chitosan-containing and chitosan-coated liposomes, respectively. Jøraholmen et al. have previously proven that chitosan-coated liposomes exhibit antimicrobial activity against *S. aureus*, even when their corresponding non-coated liposomes did not possess any activities (Jøraholmen et al., 2020).

We were also interested in the activity of plain and chitosan-comprising vesicles with CHX. The plain CHX-lipid carriers reduced the bacterial survival by 99.4%; even more than chitosan-vesicles without CHX. However, the combination of chitosan and CHX in vesicles demonstrated the strongest antimicrobial activity. Chitosan-containing CHX-liposomes completely eradicated *S. aureus*, while the bacterial survival after treatment with chitosan-coated CHX-liposomes was reduced to only 0.03%. These results are in agreement with the results from our previous study where we utilized one-pot method for the production of vesicles; however, chitosan of different M_w_ (higher M_w_) was utilized in that study (Hemmingsen et al., 2021b). Wang et al. also demonstrated improved antimicrobial activity against *S. aureus* of cinnamaldehyde, a MAA, when the compound was entrapped in chitosan-coated liposomes. Furthermore, they also demonstrated that the mechanism behind this action was membrane disruption (Wang et al., 2021). In another study, Hassan et al., proved lowered MIC and faster antimicrobial action of vancomycin when it was entrapped in lipid-chitosan hybrid vesicles. They also established that the effect was due to membrane destruction. Moreover, the authors demonstrated eradication of pre-formed MRSA biofilms (Hassan et al., 2020). These encouraging results highlight the potential of systems combining lipid-based vesicles and chitosan for successful microbial eradication.

## Conclusion

In an era of lowered microbial susceptibility to conventional antimicrobial compounds and higher prevalence of chronic wounds, often with high microbial burden, innovative strategies for microbial eradication and improved wound healing are crucially needed. We proposed that combinations of lipid-based vesicles and chitosan could serve as promising delivery systems for MAAs, such as CHX, as confirmed by successful bacterial eradication of the common skin pathogen *S. aureus*. Indeed, we showed that both chitosan-containing liposomes and chitosan-coated liposomes destined to treat infected wounds could successfully improve antimicrobial activity of CHX against *S. aureus*, highlighting their potential in antimicrobial wound therapy.

## Data availability statement

The raw data supporting the conclusions of this article will be made available by the authors, without undue reservation.

## Author contributions

LH and NŠ-B: conceptualization, formal analysis, and writing—original draft preparation. LH, KJ, PB, MJ, and NŠ-B: methodology. LH, KJ, and NŠ-B: validation. LH and PP: investigation and data curation. MN, MJ, and NŠ-B: resources. LH, PP, KJ, PB, MN, MJ, and NŠ-B: writing—review and editing. LH: visualization. NŠ-B: supervision, project administration, and funding acquisition. All authors contributed to the article and approved the submitted version.

## Funding

UiT The Arctic University of Norway, Norway funded this study (project no. 235569). The publication fund of UiT The Arctic University of Norway funded the publication charges of this article.

## Conflict of interest

The authors declare that the research was conducted in the absence of any commercial or financial relationships that could be construed as a potential conflict of interest.

## Publisher’s note

All claims expressed in this article are solely those of the authors and do not necessarily represent those of their affiliated organizations, or those of the publisher, the editors and the reviewers. Any product that may be evaluated in this article, or claim that may be made by its manufacturer, is not guaranteed or endorsed by the publisher.

## Abbreviations

AMR: Antimicrobial resistance
CCK-8: Cell counting kit-8
CFU: Colony-forming units
CHX: chlorhexidine
DMEM-hg: Dulbecco’s Modified Eagle Medium high glucose
EE: Entrapment efficiency
FBS: Fetal bovine serum
LPS: Lipopolysaccharide
MAA: Membrane-active antimicrobial
M_w_: Molecular weight
NO: Nitric oxide
PBS: Phosphate buffered-saline
PEG: Polyethylene glycol
PI: Polydispersity index
RMPI: Roswell park memorial institute medium
